# Taurine Supplementation Enhances the Resistance of *Litopenaeus vannamei* Postlarvae to Low-Salinity Stress

**DOI:** 10.3390/biology14081082

**Published:** 2025-08-19

**Authors:** Huaichi Wang, Xinyue Du, Jiahong Zou, Mengya Wang, Yan Lei, Bin Zhang, Yongzhen Zhao, Linyuan Jiang, Xiaohan Chen, Qingchao Wang

**Affiliations:** 1 Key Laboratory of Aquacultural Facility Engineering (Ministry of Agriculture and Rural Affairs), College of Fisheries, Huazhong Agricultural University, Wuhan 430070, China; wanghuaichi@webmail.hzau.edu.cn (H.W.); duxinyue@webmail.hzau.edu.cn (X.D.); zjiahong@webmail.hzau.edu.cn (J.Z.); wangmengya@webmail.hzau.edu.cn (M.W.); 2 China (Guangxi)-ASEAN Key Laboratory of Comprehensive Exploitation and Utilization of Aquatic Germplasm Resources, Ministry of Agriculture and Rural Affairs, Guangxi Key Laboratory of Aquatic Genetic Breeding and Healthy Aquaculture, Guangxi Academy of Fishery Sciences, Nanning 530021, China; sckxydb@163.com (Y.L.); zhangb41508@163.com (B.Z.); fisher1152002@126.com (Y.Z.); jianglinyuan@sina.com (L.J.); chnxhn@163.com (X.C.)

**Keywords:** *Litopenaeus vannamei*, osmoregulation, Na^+^/K^+^-ATPase (NKA), taurine, low-salinity culture, transcriptomic analysis

## Abstract

In aquaculture, Pacific white shrimp (*Litopenaeus vannamei*) growth in low-salinity waters is limited by osmoregulatory stress; therefore, improving resistance to low-salinity stress via nutritional modulation is key. In the present study, shrimp postlarvae were provided with a taurine supplement under low-salinity stress, and then the survival rate, the histology, the Na^+^/K^+^-ATPase (NKA) expression pattern and transcriptomic sequencing were investigated to evaluate the postlarval responses. The results showed that the postlarva survival rate in low-salinity water was only 61.11%, which is significantly lower than that for postlarvae reared in saline water (92.67%). However, taurine supplementation significantly increased the postlarva survival rate in low-salinity culture to 76.67% and also increased the shrimp body length. Moreover, immunofluorescence and enzyme activity assays indicated that taurine alleviated NKA overactivation in the shrimp postlarvae under low-salinity stress. Furthermore, a GO enrichment analysis of differentially expressed genes suggested that the overactivation of hormone and receptor signaling under low-salinity stress was significantly downregulated after taurine supplementation. On the other hand, taurine supplementation may promote epithelial cell proliferation in shrimp postlarvae by negatively regulating the Wnt signaling pathway. These findings suggest that taurine may enhance the shrimp postlarval osmoregulatory capacity, thereby improving their ability to acclimatize to low-salinity environments.

## Simple Summary

Shrimp (*Litopenaeus vannamei*) aquaculture in low-salinity waters is restrained by osmoregulatory stress, and taurine serves as an important osmolyte in multiple biological processes. In the present study, taurine was supplemented to shrimp postlarvae reared in low-salinity waters, and then shrimp survival, histology, and Na^+^/K^+^-ATPase (NKA) expression pattern were assessed and transcriptomic sequencing was conducted to evaluate its role in regulating postlarvae resistance capacity against low-salinity stress. The results showed that taurine supplementation significantly promoted the shrimp postlarvae survival rate in low-salinity waters from 61.11% to 76.67% and increased shrimp body length. Taurine also alleviated the low-salinity-stress-induced over-activation of NKA protein and its enzyme activity in shrimp postlarvae. Transcriptome analysis showed that compared to the control group, low-salinity stress induced 454 upregulated and 283 downregulated genes, whose numbers decreased to 396 and 133 after taurine supplementation. Further enrichment analysis of these differentially expressed genes indicated that the low-salinity-stress-induced over-activation of hormone and receptor signaling were mitigated after taurine supplementation, while taurine promoted the proliferation of shrimp postlarvae epithelial cells by negatively regulating the Wnt pathway. Our study identified the key role of taurine in enhancing the resistance capacity of shrimp postlarvae against low-salinity stress, thus promoting the continual development of shrimp aquaculture.

## 1. Introduction

The Pacific white shrimp (*Litopenaeus vannamei*) is an important aquaculture species with high nutritive and commercial value and is cultured around the world [1]. *L. vannamei* has multiple advantages for aquaculture, including broad temperature and salinity adaptability, a short growth cycle, low nutritional requirements and strong disease resistance, which have contributed to its widespread distribution [2]. Usually, shrimp is reared in saline water environments as a tropical marine species; however, viral and parasitic diseases significantly decrease the success rate of traditional marine shrimp farming [3]. Although low-salinity shrimp culture technology has been successfully developed and rapidly disseminated, shrimp aquaculture in low-salinity water systems is continuously challenged by a lower yield per unit area and altered muscle quality [4]. All of these issues are closely related to osmotic stress caused by low-salinity conditions; therefore, the osmoregulatory mechanism in shrimp under low-salinity stress needs to be elucidated. Then, nutritional modulatory methods can be developed to enhance the osmoregulatory capacity and yield of shrimp freshwater culture.

*L. vannamei* is a typical euryhaline crustacean and exhibits a strong ability to adapt to water environments of varying salinities via osmoregulation, which has facilitated its widespread geographical distribution. In seawater, euryhaline crustaceans act as osmoconformers, with the osmotic pressure of their hemolymph directly reflecting that of the external environment [5]. If water salinity drops below 26‰, this shrimp will activate hyper-osmoregulatory mechanisms, which are reliant on a suite of ion transport carriers and enzymes. Of these, Na^+^/K^+^-ATPase (NKA), which establishes electrochemical gradients for ion transport into the hemolymph, and carbonic anhydrase (CA), which generates H^+^ and HCO_3_^−^ in the cytoplasm, have been most extensively studied [6]. NKA serves as the core functional protein for osmoregulation in *L. vannamei* and is primarily distributed in tissues such as the gills and hindgut, where it actively transports sodium and potassium ions to maintain osmotic balance across cellular membranes [7]. NKA is composed of an α subunit, β subunits, and regulatory subunits; the α subunit harbors binding sites for Na^+^, K^+^ and ATP and is responsible for primary catalytic and ion transport functions, while the β subunit plays a critical role in stabilizing the folding of the α subunit [8]. Studies show that the posterior gills of euryhaline crustaceans exhibit peak NKA enzyme activity when transferred to low-salinity environments, which is associated with transcriptional changes in *NKAα* mRNA [9]. In order to reveal the physiological mechanisms underlying these osmoregulatory adaptations, several studies have also evaluated the role of hormones and endocrine glands in osmoregulation [10]. In *Metapenaeus monoceros*, eyestalk extirpation causes a substantial change in Na^+^ and K^+^ ions in the hemolymph [11]. Additionally, Diwan and Laxminarayana [12] studied the influence of various neuroendocrine centers on the osmotic concentration of hemolymph. Eyestalk neuroendocrine factors are believed to help maintain or regulate the hemolymph’s ionic concentration. Studies in Artemia have even reported the occurrence of five types of neurosecretory cells in the entire neuroendocrine system in this genus [13].

Besides the involvement of NKA and endocrine regulation of hemolymph osmotic pressure, crustacean tissues also contain osmolytes like glycine betaine and free amino acids for isosmotic intracellular regulation (IIR). Initially, the inorganic ion levels (primarily of Na^+^) are adjusted to modulate the cell volume during salinity flux, which is followed by the accumulation or degradation of specific osmolytes to restore the intracellular osmotic balance and normal cell volume [14]. Under low-salinity conditions, shrimp require substantial energy and osmolytes to adapt to osmotic changes, significantly altering their nutritional requirements. Studies have reported osmotic regulatory roles for free amino acids in muscle, with their concentrations in hemolymph and muscle shifting post-salinity alteration [15]. In one study, the taurine content in razor clam *Sinonovacula constricta* hemolymph significantly increased after hyposalinity stress, implying that taurine has a potential role in osmoregulation in aquatic animals [16]. In fact, taurine, a critical functional amino acid, participates in numerous biological regulatory processes including heart rhythm modulation, nerve impulse transmission, cell proliferation, and bile acid synthesis—functions closely associated with ion transport and protein phosphorylation [17]. Taurine can also modulate Na^+^ transport through shared β-amino acid transporters and by altering Na^+^ channel activation states and can also modulate K^+^ transport via ATP-sensitive K^+^ channels [18]. Unlike glycine betaine, free amino acids also provide energy during the process of low-salinity adaptation. After being transported into mitochondria via TauT (taurine transporter), taurine not only provides energy through amino acid catabolism but also acts as a signaling molecule to activate downstream amino acid-sensing pathways (e.g., the mTOR and AAR pathways), phosphorylating target proteins like S6 to regulate cellular energy metabolism and protein synthesis [19].

Early studies in Pacific white shrimp reported the optimal taurine level to be 0.168% of the dry diet when salinity was at 29–30‰ [20], but it was 0.437–0.579% [21] and 0.57–0.60% [22] when salinity was at 5.5–6.0‰. Therefore, it is understandable that shrimp require substantial energy and osmolytes to adapt to osmotic changes under low-salinity conditions, thus significantly altering their nutritional demands. Further research is required on the osmoregulatory function of taurine under low-salinity conditions in shrimp at different growth periods. With developments in the fields of molecular biology and genomics in recent years, studies have employed transcriptome and proteome sequencing technologies to screen differentially expressed genes (DEGs) and proteins in *L. vannamei* under varying salinity levels, aiming to identify associated signaling pathways [23]. The different categories of genes that have been reported are those controlling osmotic stress sensing, signal transduction, osmotic stress tolerance, ion transportation, active ion exchange and cell volume regulation [24]; their discovery has helped reveal the mechanisms of osmoregulatory adaptations at the genome level. However, current research mainly focuses on shrimp juveniles and adults, with little information revealed on osmoregulatory regulation during the early developmental stages, including in shrimp postlarvae, which urgently requires further in-depth investigation. In the present study, transcriptomic sequencing, along with shrimp postlarval survival, growth, histology, and osmoregulation-related enzyme activity assays, was conducted to explore taurine’s functions in shrimp postlarvae under low-salinity stress.

## 2. Materials and Methods

### 2.1. Ethical Statement

The shrimp rearing experiments and related procedures were approved by the Animal Experimentation Committee of Huazhong Agricultural University (Approval No. HZAUFI-2017-001). All experimental protocols complied with institutional guidelines for the ethical use of aquatic organisms.

### 2.2. Experimental Design

Healthy white shrimp postlarvae from the same breeding batch (salinity of 18‰) with uniform size were cultured in indoor experimental tanks with water of different salinities and divided into three groups: control group (C, 18‰), low-salinity group (L, salinity gradually reduced from 18‰ down to 4‰ at a rate of 2‰ per day), and low-salinity + taurine group (T, similar salinity to L group). A commercial diet (41% protein, 4.5% fat, 4.5% fiber and 16% ash, from Evergreen Feed, Zhanjiang, China) was used as the basal diet to feed shrimp postlarvae in the C and L groups. The experimental diet in the T group was formulated by dissolving taurine in water and then spraying this on the basal feed pellets (0.3% of dry feed weight). Each treatment consisted of three replicates with 300 postlarvae in each experimental aquarium. Water temperature, dissolved oxygen and pH were maintained within ranges of 29 ± 1 °C, 7.1 ± 0.4 mg/L and 7.4 ± 0.6, respectively. At the end of the one-week feeding trial, the shrimp postlarvae were collected to calculate the survival rate and body length. Some postlarva samples were fixed in paraformaldehyde or stored at −80 °C for subsequent analysis.

### 2.3. Histological Sample Preparation

Shrimp specimens were fixed by immersion in 4% paraformaldehyde solution for over 24 h at room temperature. Following fixation, shrimp specimens underwent graded alcohol dehydration through a series of ethanol solutions: 60% for 4 h, 70% overnight (8–10 h), 80% for 2 h, 90% for 2 h, 95% for 2.5 h and 100% for 1 h. The dehydrated samples were then cleared in an ethanol/xylene (1:1) solution for 10 min followed by xylene immersion twice (7 min each). For paraffin infiltration, shrimp specimens were placed in molten paraffin wax at 60 °C for 1 h, which was repeated three times. Embedding was performed by carefully transferring the infiltrated tissues to embedding molds, orienting them appropriately, and filling the molds with molten paraffin before allowing them to solidify at room temperature. The resulting paraffin blocks were sectioned continuously at a thickness of 5 μm; these sections were subsequently dewaxed in xylene and rehydrated through a graded alcohol series prior to staining with hematoxylin and eosin (H&E). Finally, the stained sections were examined under a light microscope (Olympus, DP72) equipped with a camera (Model E600, Tokyo, Japan) and CellSens Standard Software (version 4.1, Olympus Corporation, Tokyo, Japan) for image acquisition.

### 2.4. Immunofluorescence Staining

Wax and xylene were removed from the paraffin sections, which were then subjected to antigen retrieval with 1% EDTA-2Na solution in a microwave oven and allowed to cool completely to room temperature. The slides were washed twice with PBS for 5 min each. After circling the tissues with a histochemical pen, they were blocked with protein blocking solution for 30 min, which was then removed by washing with phosphate-buffered saline (PBS). Diluted mouse-derived NKA primary antibody (ABclonal, Wuhan, China) was then applied to the tissue sections; they were incubated overnight at 4 °C and then washed four times with PBS to remove unbound antibodies. Diluted goat anti-mouse secondary antibody was subsequently added to the sections, which were incubated at room temperature for 45 min, and washed another four times with PBS. Finally, cell nuclei were stained with DAPI for 8 min at room temperature. After washing twice with PBS, excess water in the tissues was absorbed with filter paper, and anti-fluorescence quenching mounting medium was applied before coverslipping. Fluorescence signals were measured using an Olympus BX53 fluorescence microscope (Olympus Corporation, Tokyo, Japan) and analyzed with the corresponding imaging system.

### 2.5. NKA Enzyme Activity Analysis

Shrimp postlarval samples from different experimental groups stored at −80 °C were accurately weighed and homogenized in 9 volumes of physiological saline using a mechanical homogenizer in an ice bath. The homogenate was centrifuged at 2500 r/min for 10 min, and the supernatant was collected. Na^+^/K^+^-ATPase (NKA) activity was determined in 10% homogenate supernatant using the phosphomolybdate method. Protein concentration was first measured using the Coomassie Brilliant Blue assay kit (W067, Nanjing Jiancheng Bioengineering Institute, Nanjing, China). NKA activity was then quantified with the ultra-micro Na^+^/K^+^-ATPase assay kit (A070-2, Nanjing Jiancheng Bioengineering Institute). One unit of enzyme activity (U) was defined as the amount of enzyme required to hydrolyze 1 μmol of ATP to ADP per hour per milligram of postlarval tissue protein, with inorganic phosphate release as the measured product. Three biological replicates were conducted for each group.

### 2.6. RNA Extraction and Transcriptomic Sequencing

Total RNA was extracted from pooled whole-body postlarval shrimp samples (20 shrimp postlarvae per experimental group) using TRIzol reagent (Magen, Guangzhou, China). Following the manufacturer’s instructions, samples were homogenized with steel beads, and RNA purity/concentration was assessed by NanoDrop 2000 spectrophotometry (Thermo Fisher Scientific Inc., Wilmington, DE, USA), with the RIN of RNA determined using an Agilent Bioanalyzer 4150 system (Agilent Technologies, Palo Alto, CA, USA). Only qualified mRNA samples from different groups were fragmented and used for library construction, which was followed by transcriptome sequencing on the Illumina NovaSeq 6000 platform. Statistical analysis and quality assessment, including measurement of base composition, quality scores, and error rate distribution, were performed on the raw sequencing data.

Raw data in fastq format were first processed through in-house perl scripts. In this step, adapter sequences and low-quality reads were removed using fastp software v1.0.1 (https://github.com/OpenGene/fastp (accessed on 17 June 2025)) to obtain high-quality clean reads, followed by re-evaluation of sequencing statistics and quality metrics. The quality-controlled reads were aligned to the reference genome (*L. vannamei*, NCBI Assembly ID: GCA_018831695.1) using HiSat2 v2.2.1 (http://ccb.jhu.edu/software/hisat2/index.shtml (accessed on 1 July 2021)). Assessing the quality of the alignment results involved sequencing saturation analysis, gene coverage evaluation, and analysis of read distribution across genomic regions (e.g., exons, introns, and intergenic regions) and chromosomes.

The levels of gene and transcript expression were quantified using RSEM software v1.3.3 (http://deweylab.github.io/RSEM/ (accessed on 14 February 2020)) for subsequent differential expression analysis across samples. Differential gene expression analysis was performed with DESeq2 v1.44.0 (http://bioconductor.org/packages/stats/bioc/DESeq2 (accessed on 17 August 2025)) to identify differentially expressed genes (DEGs). Significant DEGs were identified using the thresholds of a false discovery rate (FDR) of <0.05 and absolute log2 fold change (|log2FC|) of ≥1. The Gene Ontology (GO) database (http://geneontology.org/ (accessed on 22 July 2025)) was utilized to classify genes based on their involvement in biological processes, cellular components, and molecular functions, with subsequent GO annotation of DEGs. The Kyoto Encyclopedia of Genes and Genomes (KEGG) database (https://www.genome.jp/kegg/ (accessed on 1 August 2025)) was employed to categorize DEGs into pathways or functional classes, which was followed by annotation. GO enrichment analysis was performed using GOATOOLS v1.3.0 (https://github.com/tanghaibao/goatools (accessed on 2 February 2025)), while KEGG pathway enrichment analysis was conducted with the Python SciPy package v1.13.0 (https://scipy.org/install/ (accessed on 27 July 2025)). Fisher’s exact test was applied for statistical evaluation, with multiple testing correction performed via the Benjamini–Hochberg method. Terms with an adjusted *p*-value (FDR) ≤ 0.05 were considered significantly enriched.

### 2.7. Confirmation of the Illumina Sequencing Profiles by Quantitative Real-Time PCR

To verify the sequencing results, ten genes were randomly selected for quantitative real-time PCR. Shrimp postlarva RNA was collected using the TRIzol reagent, as described above. cDNA was synthesized from 1 μg of total RNA using the reverse transcriptase kit (Invitrogen, Carlsbad, CA, USA) with oligo dT primers following the manufacturer’s instructions. Quantitative real-time PCR was conducted on a 7500 Real-time PCR system (Applied Biosystems, Foster City, CA, USA) using the Eva Green 2 × qPCR Master mix (ABM Inc., Richmond, BC, Canada). Each PCR was performed with triplicate samples and the cycling conditions were set to 30 s at 95 °C, 1 s at 95 °C and 10 s at 58 °C for 40 cycles. In addition, a melt curve analysis was performed after amplification to verify the accuracy of each amplicon. The shrimp *β-actin* gene was chosen as the internal reference gene. Then, the relative abundance of target genes was calculated using the 2^−ΔΔCt^ method. All primers used in the present study are shown in Supplementary Table S1.

### 2.8. Statistical Analysis

Results were analyzed by one-way ANOVA, and a homogeneity of variance test was conducted to ensure that variance was homogeneous. Tukey’s test was utilized to compare individual means, with differences considered significant at *p* < 0.05.

## 3. Results

### 3.1. Taurine Enhances Survival and Growth of Litopenaeus vannamei Postlarvae Under Low-Salinity Stress

**Figure 1:**
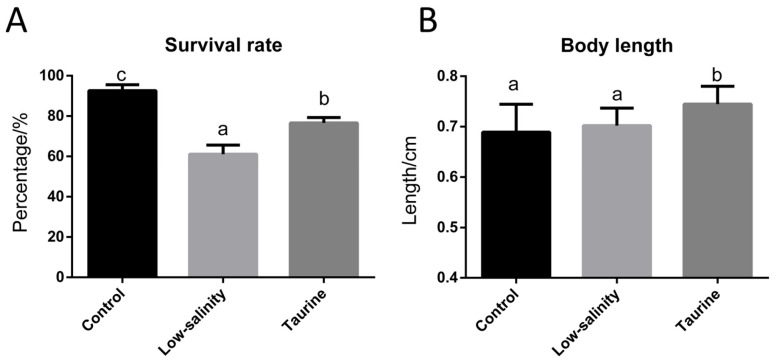
The survival rate (**A**) and body length (**B**) of *L. vannamei* postlarvae reared in saline water, low-salinity water and low-salinity water + taurine supplementation. Mean values with different letters indicate significant differences among groups (*p* < 0.05).

As shown in Figure 1A, the survival rate of the shrimp postlarvae cultured in saline water was 92.67%, while it decreased to 61.11% under low-salinity conditions. However, taurine supplementation in the low-salinity environment significantly improved the survival rate of the shrimp postlarvae to approximately 76.67%. Figure 1B reveals that the shrimp body length showed no significant difference between the low-salinity and saline water groups, while taurine supplementation significantly increased the shrimp postlarva body length, which was significantly higher than that in the C and L groups.

### 3.2. Taurine Protected the Histological Structure of L. vannamei Postlarvae Under Low-Salinity Stress

**Figure 2:**
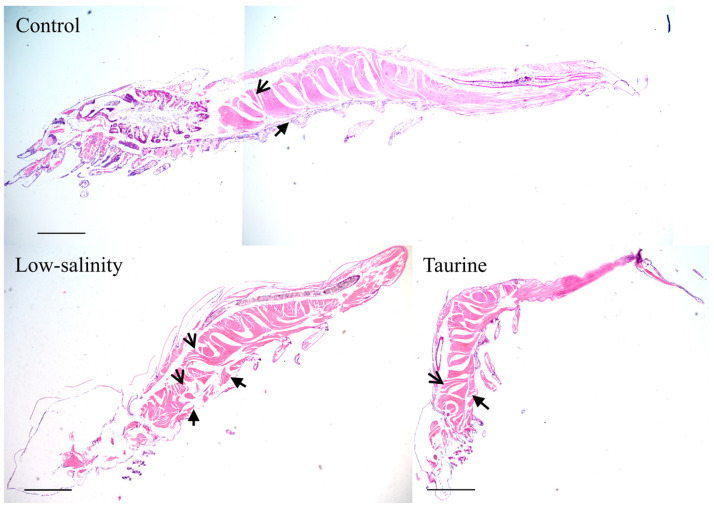
The merged histological structure of *L. vannamei* postlarvae reared in saline water, low-salinity water, and low-salinity water with taurine supplementation. 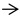 indicates intermuscular spaces; 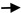 indicates the demarcation between visceral organs. Scale bar: 500 μm.

Figure 2 reveals the distinct morphological differences in the whole shrimp postlarva histological sections across the three treatment groups. In saline water, the shrimp in the C group exhibited intact structural morphology with clearly defined tissue organization and sharp boundaries between anatomical components. In contrast, low-salinity stress caused a noticeable expansion of intermuscular spaces and blurred demarcation between visceral organs in L group. Taurine supplementation led to significant restorative effects: muscle fibers regained compactness compared to those in the low-salinity group, and organ boundaries became more distinct than those in the L group.

### 3.3. Taurine Alleviated the Overactivation of Na^+^/K^+^-ATPase (NKA) in Litopenaeus vannamei Postlarvae Under Low-Salinity Stress

**Figure 3:**
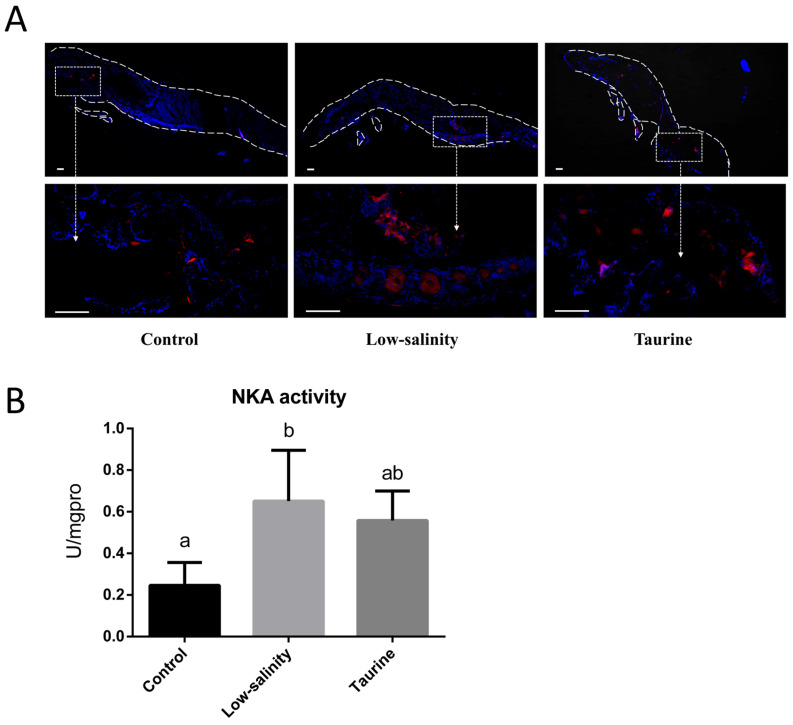
The in situ Na^+^/K^+^-ATPase (NKA) expression (**A**) and NKA enzyme activity (**B**) in *L. vannamei* postlarvae reared in saline water, low-salinity water, and low-salinity water with taurine supplementation (one unit of NKA enzyme activity (U) was defined as the amount of enzyme required to hydrolyze 1 μmol of ATP to ADP per hour per milligram of postlarval tissue protein, with inorganic phosphate release as the measured product). Mean values with different letters indicate significant differences among groups (*p* < 0.05). In (**A**), the blue color represents the cell nuclei, while the red color indicates the NKA protein. Scale bar: 100 μm.

The in situ NKA protein expression in the shrimp postlarvae was evaluated via an immunofluorescence assay using an NKA-specific antibody. The results indicated that low-salinity stress induced elevated NKA protein expression in shrimp postlarvae compared to that in the control group, with intensified red fluorescence signals. However, taurine supplementation significantly alleviated NKA over-expression under low-salinity stress (Figure 3A).

Similarly, the Na^+^/K^+^-ATPase (NKA) enzymatic activity in the three experimental groups was also analyzed. As shown in Figure 3B, the shrimp postlarvae in the L group exhibited significantly higher NKA activity compared to that in the control group, indicating that osmoregulatory stress was induced in shrimp to adapt to environmental osmotic changes. Interestingly, the NKA activity in the shrimp postlarvae under low-salinity stress after taurine supplementation showed no significant difference with that in the control group.

### 3.4. Transcriptomic Sequencing Suggested the Protective Mechanism of Taurine in Shrimp Postlarvae Under Low-Salinity Stress

**Figure 4:**
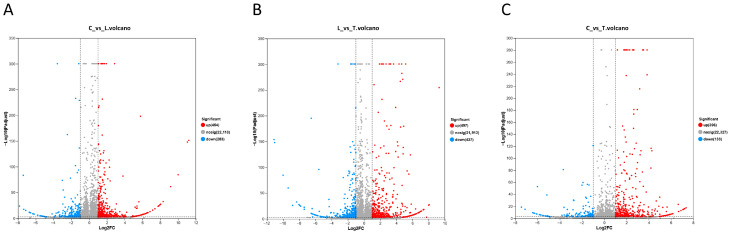
Differential expression analysis of transcriptomic profiling in *L. vannamei* postlarvae reared in three groups. (**A**) Volcano plot of transcript expression in *L. vannamei* reared in low-salinity water (L) compared with control (C). (**B**) Volcano plot of transcript expression in *L. vannamei* reared in low-salinity water + taurine supplementation (T) compared with L group. (**C**) Volcano plot of transcript expression in *L. vannamei* reared in low-salinity water + taurine supplementation (T) compared with C group.

In this study, pooled RNA samples extracted from the whole bodies of 20 shrimp postlarvae per experimental group were sent to Majorbio Biotechnology Co., Ltd. (Shanghai, China) for high-throughput sequencing analysis, with each group generating over 87,316,458 raw reads. As presented in Supplementary Table S2, the Q30 base percentage for all of the samples was no less than 97.02%, and the GC content ranged between 44.82% and 46.11%, meeting high-quality standards. The DEGs in the three experimental groups are shown in Figure 4 and Supplementary Table S3. The shrimp postlarvae in the low-salinity group exhibited 737 differentially expressed genes (454 upregulated and 283 downregulated) compared to the control group. However, the shrimp postlarvae in the low-salinity + taurine group only showed 529 differentially expressed genes (396 upregulated and 133 downregulated) compared to the control group. Additionally, there were 497 more upregulated and 437 more downregulated genes in the T group compared to the L group. The RNA-seq data were deposited in the NCBI Sequence Read Archive (SRA) under the permanent accession number PRJNA1295440.

**Figure 5:**
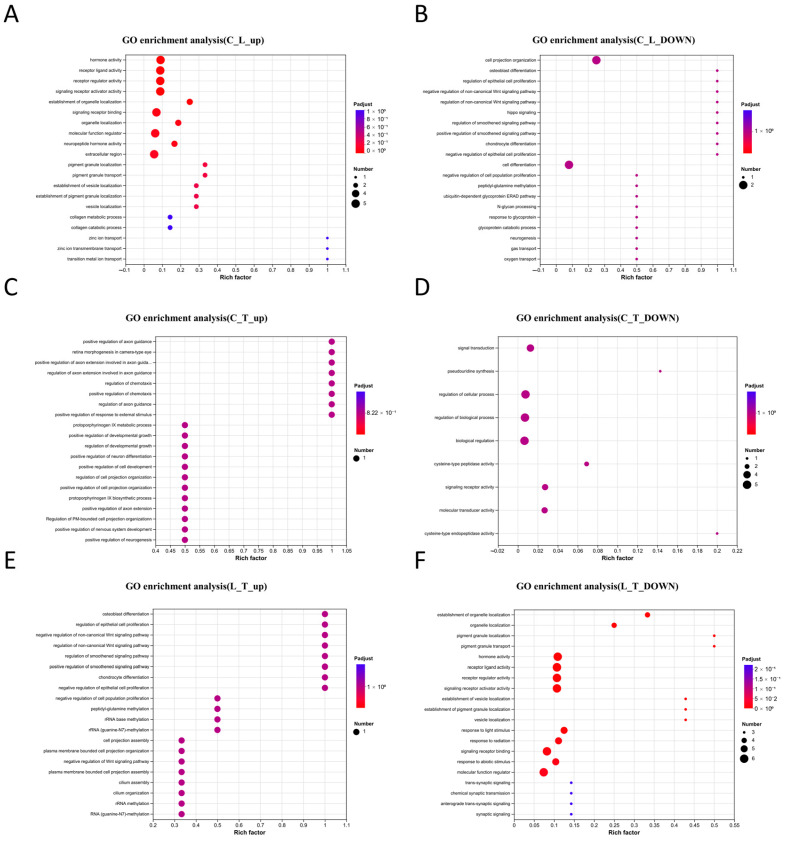
Statistics of GO enrichment of the upregulated and downregulated genes in three groups of *L. vannamei* postlarvae. GO enrichment was analyzed based on adjusted *p* < 0.05. (**A**) GO enrichment of the upregulated genes in the L group vs. the C group. (**B**) GO enrichment of the downregulated genes in the L group vs. the C group. (**C**) GO enrichment of the upregulated genes in the T group vs. the C group. (**D**) GO enrichment of the downregulated genes in the T group vs. the C group. (**E**) GO enrichment of the upregulated genes in the T group vs. the L group. (**F**) GO enrichment of the downregulated genes in the T group vs. the L group.

Figure 5 demonstrates the GO enrichment analysis results for the DEGs in the three experimental groups. Compared to the control group, the upregulated genes in the L group were mainly enriched in hormone activity, receptor ligand activity, receptor regulator activity, signal receptor activator activity, signal receptor binding, molecular function regulation, collagen metabolic processes and zinc ion transport (Figure 5A). On the other hand, the genes downregulated in the L group with respect to the C group were mainly enriched in epithelial cell proliferation regulation, positive and negative regulation of the non-canonical Wnt signaling pathway, the Hippo signaling pathway, negative regulation of epithelial cell proliferation, negative regulation of cell population proliferation, gas transport and oxygen transport (Figure 5B). As shown in Figure 5C, compared to the C group, the upregulated genes in the T group were mainly enriched in the regulation of chemotaxis, positive regulation of the response to external stimuli, positive regulation of developmental growth, positive regulation of cell development and positive regulation of nervous system development when compared to the saline group, while the downregulated genes were mainly enriched in signal transduction, regulation of cellular processes, regulation of biological processes, signal receptor activity and cysteine-type endopeptidase activity. Moreover, compared to the L group, the T group showed upregulated genes enriched in the regulation of epithelial cell proliferation, negative regulation of epithelial cell proliferation, negative regulation of the non-classical Wnt signaling pathway, negative regulation of cell population proliferation and peptidyl-glutamine methylation compared with the low-salinity group, while the T group showed downregulated genes enriched in hormone activity, receptor ligand activity, receptor regulator activity, signal receptor activator activity, signal receptor binding, the response to light stimuli and the response to abiotic stimuli.

**Figure 6:**
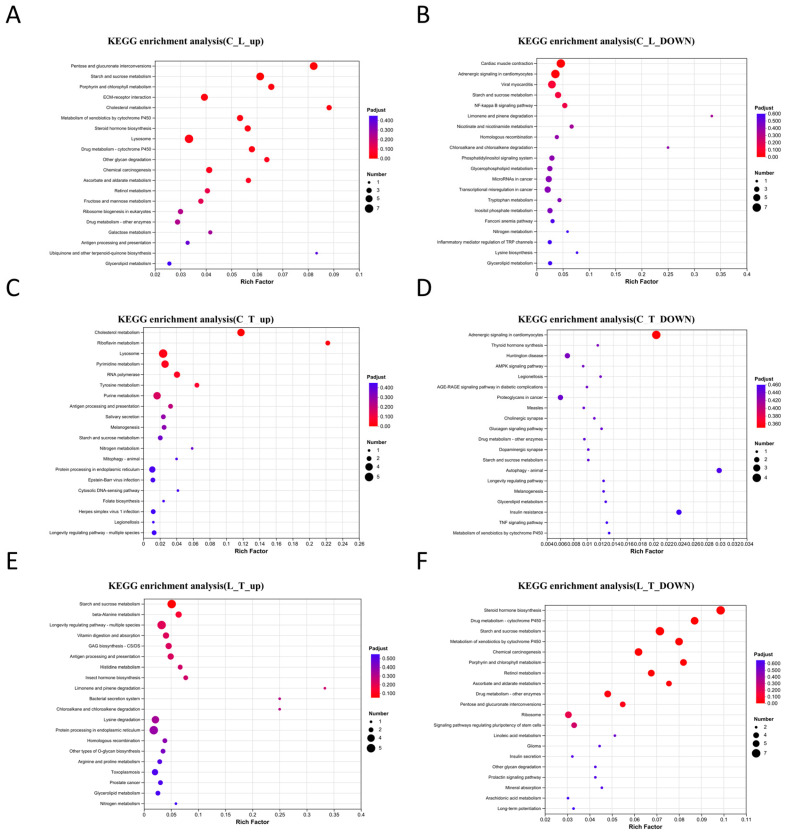
Statistics of KEGG enrichment of the upregulated and downregulated genes in three groups of *L. vannamei* postlarvae. KEGG enrichment was analyzed based on the adjusted *p* < 0.05. (**A**) KEGG enrichment of the upregulated genes in the L group vs. the C group. (**B**) KEGG enrichment of the downregulated genes in the L group vs. the C group. (**C**) KEGG enrichment of the upregulated genes in the T group vs. the C group. (**D**) KEGG enrichment of the downregulated genes in the T group vs. the C group. (**E**) KEGG enrichment of the upregulated genes in the T group vs. the L group. (**F**) KEGG enrichment of the downregulated genes in the T group vs. the L group.

The results of the KEGG enrichment analysis of the DEGs in the three experimental groups are presented in Figure 6. Compared to the control group, the upregulated genes in the L group were mainly enriched in extracellular matrix–receptor interaction, cholesterol metabolism, steroid hormone biosynthesis, and antigen processing and presentation, while the downregulated genes in the L group were mainly enriched in cardiac muscle contraction, adrenergic signaling in cardiomyocytes, viral myocarditis, and the NF-kB signaling pathway. However, in the low-salinity plus taurine group, the upregulated genes compared to the C group were mainly enriched in cholesterol metabolism, lysosomes, antigen processing and presentation, herpes simplex virus 1 (HSV-1) infection and legionellosis when compared with the saline water group, while the downregulated genes were enriched in adrenergic signaling in cardiomyocytes, thyroid hormone synthesis, legionellosis and animal autophagy. Furthermore, the upregulated genes after taurine supplementation were mainly enriched in starch and sucrose metabolism, vitamin digestion and absorption, antigen processing and presentation and protein processing in endoplasmic reticulum when compared with the L group, while the downregulated genes were mainly enriched in steroid hormone biosynthesis, ascorbate and aldarate metabolism and ribosomes.

### 3.5. Validation of DGE by QPCR

**Figure 7:**
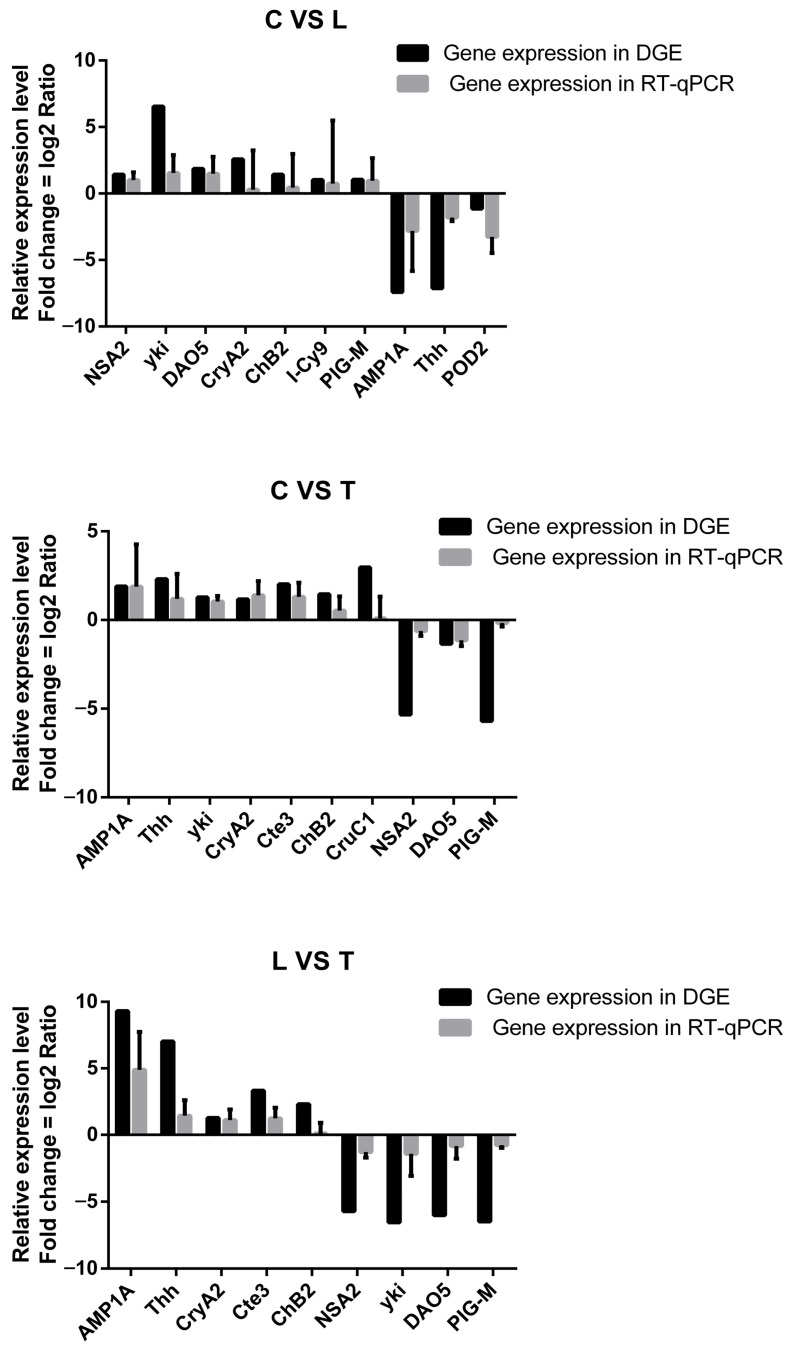
A comparison of relative fold change in gene expression in the DGE and RT-qPCR results among the C, L and T groups in *Litopenaeus vannamei* postlarvae. The transcript expression levels of the selected genes were each normalized to that of the *β-actin* gene.

qRT-PCR was conducted to validate the differentially expressed genes identified by DGE, in which the melting curve analysis certified a single product for all of the tested genes. Fold changes in qRT-PCR were compared with the DGE expression analysis results (Figure 7). In general, the DGE results aligned with the qRT-PCR results, indicating the reliability and accuracy of the DGE analysis.

## 4. Discussion

*L. vannamei* can survive in brackish water as well as freshwater regions due to its euryhaline characteristics (0.5 to 45 ppt) [25]. In aquaculture, rainstorms, high evaporation rates and natural episodic fluxes of freshwater result in ambient salinity fluctuations, which significantly affect shrimp’s ecophysiological performance [26,27]. The saline tolerance of juvenile and adult shrimp depends on several factors including the salinity range, developmental stage and whether exposure occurs through direct or gradual transfer [28]. Although plenty of studies have identified the transcriptomic and proteomic responses of juvenile and adult shrimp [29,30] during salinity changes, little information is known about the responses during the early life stages, including in postlarvae. Moreover, improving the shrimp postlarva performance during salinity fluxes via nutritional modulation methods is key [31]. Previous studies in tilapia (*O. mossambicus*) have identified the accumulation of organic osmolytes in tissues and the upregulation of taurine transporter mRNA in response to salinity changes, indicating that taurine may have some role in osmoregulatory acclimation in this species [32]. Our study evaluated the survival, growth, histology, NKA expression, enzyme activity and transcriptomic responses of shrimp postlarvae under low-salinity stress and after taurine supplementation.

### 4.1. Taurine Significantly Improves Postlarval Survival and Repairs Tissue Damage Under Low-Salinity Stress

Salinity is one of the most important factors that affect the physiology as well as growth of shrimp [28]. As mentioned above, low-salinity exposure occurring through direct or gradual transfer significantly affects the saline tolerance of juveniles and adults [28]. In our study, in order to simulate an extremely saline environment, the water salinity for the low-salinity stress (L) group was decreased from 18‰ to 4‰ in seven days. As shown in Figure 1, the survival rate of the shrimp postlarvae in the L group decreased to 61.11% compared to 92.67% in the control (C) group, indicating that shrimp larvae cannot tolerate such rapid salinity changes. Early studies showed that shrimp (3.0 ± 0.23 g) exposed to a sudden decrease in salinity from 30 to 0.3 ppt displayed a significantly lower survival rate (11.91%), and those experiencing rapid adaptation showed a higher survival rate (86.67%), but this was still lower than that in the control group (100%) [30]. Although the full length of the shrimp postlarvae showed no significant difference between the C and L groups, the histology of the shrimp postlarvae in the L group showed a noticeable expansion of intermuscular spaces and blurred demarcation between visceral organs; this was significantly different from the histology of the shrimp postlarvae in the C group, which exhibited intact structural morphology with clearly defined tissue organization and sharp boundaries between anatomical components. Early studies showed that shrimp (3.0 ± 0.23 g) exposed to a sudden decrease in salinity from 30 to 0.3 ppt also displayed histological changes and lesions, including a disordered hepatopancreas tubule structure, sloughing of tubule epithelial cells, and inter-tubular hemocyte infiltration [30].

In order to improve shrimp postlarva performance under low-salinity stress, the postlarvae were given a taurine supplement. Taurine has been reported to increase the activities of intestinal and hepatopancreatic lipase, protease, and amylase, thereby improving the specific growth rate and feed conversion efficiency in mud crab *Scylla paramamosain* [33]. Taurine supplementation has been reported to enhance the growth performance of GIFT reared in ion-imbalanced low-salinity water [34]. In shrimp (1.59 ± 0.03 g) reared at 28 °C, taurine supplementation with 0.6 to 0.8% significantly increased the FBW, WGR and PER (*p* < 0.05) [22]. Moreover, taurine supplementation in mostly plant-based diets also enhanced the cholesterol utilization and growth of Pacific white shrimp postlarvae (2 mg) in a biofloc system [35]. In our study, taurine supplementation significantly improved the survival rate (76.67%) of shrimp postlarvae under low-salinity stress, which is in accordance with previous studies showing that taurine can enhance immunity and antioxidant capacity [36] and also improve clam (*Ruditapes philippinarum*) performance during air exposure [37]. The full length of the shrimp postlarvae in the T group was also significantly higher than that in the other two groups. Moreover, the taurine supplementation exhibited significant restorative effects, as muscle fibers regained compactness compared to the low-salinity group, and organ boundaries became more distinct than those in the L group. All of these results suggest that taurine alleviates freshwater-induced damage in shrimp postlarvae and promotes their growth.

### 4.2. Taurine Maintains Ionic Homeostasis in Low-Salinity Environments Through NKA Regulation

Salinity variation is directly related to osmoregulation; shrimp can regulate osmolytes to control the hemolymph osmotic pressure [28]. Considering that Na^+^/K^+^-ATPase is a ubiquitous enzyme in all animal cell membranes and hydrolyzes ATP, driving the translocation of three Na^+^ out of the cytosol and two K^+^ into the cytosol [38], NKA is primarily responsible for osmotic and ionic homeostasis in crustaceans. In the present study, we evaluated the in situ NKA protein expression in shrimp postlarvae via immunofluorescence for the first time. The results showed that low-salinity stress induced both increased in situ NKA protein expression and NKA enzymatic activity in shrimp postlarvae, suggesting that osmoregulatory stress was induced in shrimp so they could adapt to environmental osmotic changes. Such results are in accordance with those of previous studies in shrimp and other crustaceans [39]. There was an observed increase in the expression of *NKAα* subunit mRNA in both the hepatopancreas and gills after transferring the shrimp from 30‰ salinity water to 7.5 ‰ salinity water [40,41]. Moreover, an increase (*p* > 0.05) in *NKAα1* mRNA was detected in the DLSW group compared to the FW group, probably owing to the imbalanced Na^+^, Cl^−^ and K^+^ ratio in the surrounding medium, which ultimately elevated the activity of the NKA pump. The change in salinity affects the expression of the *NKAα* subunit and regulates NKA activity. As a result, NKA causes cell ion flux to balance the osmotic pressure. The acclimation of shrimp to different salinities shows that the *NKAα* expression levels and enzyme activity peak after 6 h of exposure to low salinity [40]. In some crustaceans, α-subunit gene or protein expression is increased after exposure to dilute media [39].

NKA expression and enzyme activity were not only affected by salinity but were also modulated by nutrients. A previous study showed that the total and specific Na^+^/K^+^-ATPase activity in shrimp after acute salinity exposure was significantly decreased by high-HUFA diets [42]. Taurine and/or inorganic potassium as dietary osmolytes countered low-salinity stress and enhanced the growth of GIFT reared in ion-imbalanced low-salinity water. Specifically, taurine inclusion led to much larger improvement compared to K^+^ supplementation. NKA expression decreased to a level even less than that found with freshwater, suggesting that taurine is an efficient osmolyte in GIFT reared under PDLSW [34]. In this study, taurine supplementation effectively mitigated Na^+^/K^+^-ATPase (NKA) overactivity under low-salinity stress. The NKA activity of shrimp postlarvae under low-salinity stress after taurine supplementation showed no significant difference with that in the control group, indicating that taurine may function as an osmoregulatory molecule to maintain osmotic balance and facilitate acclimation to low-salinity waters, which may be responsible for enhancing survival and growth.

### 4.3. Transcriptomics Reveals Taurine Enhances Osmoregulatory Adaptation via Wnt Signaling Pathway

In order to elucidate the regulatory mechanism of low-salinity stress, transcriptomic sequencing was performed, identifying 737 differentially expressed genes (454 upregulated and 283 downregulated) in shrimp postlarvae in the low-salinity group compared to those in the control group. The GO enrichment analysis of the DEGs in the C and L groups showed that hormone- and receptor-related activity, collagen metabolism and zinc ion transport were upregulated in the L group. Specifically, the upregulated hormone- and receptor-related activity may suggest that low-salinity stress activates hormone release and receptor-mediated downstream pathways to induce osmotic regulation [43]. Moreover, enhanced collagen metabolism may be linked to barrier repair mechanisms [44], and increased zinc ion transport implies potential ROS scavenging through zinc’s cofactor function [45]. On the other hand, low-salinity stress significantly inhibited the epithelial proliferation pathways (non-canonical Wnt/Hippo signaling), which represents a protective response against osmotic stress by reducing epithelial turnover and cell migration [46]. Moreover, the decreased gas/oxygen transport in the L group may suggest decreased locomotor activity, which is responsible for the lower survival in the L group. Besides the GO enrichment, the KEGG enrichment analysis of the DEGs in the C and L groups also identified upregulated extracellular matrix–receptor interaction in the L group. Furthermore, low-salinity stress also induced upregulated cholesterol and steroid hormone biosynthesis, which appears to be an adaptive response to deal with low-salinity stress [47]. On the other hand, upregulated antigen processing and presentation but downregulated NF-kB signaling may suggest that the immune system in shrimp larvae cannot deal with environmental antigens, but they can be processed and presented [48], which may also be responsible for the lower survival rate in the L group.

Under low-salinity conditions, taurine supplementation resulted in 497 upregulated and 437 downregulated genes, suggesting taurine’s effect on gene expression. The GO enrichment analysis of the DEGs in the L and T groups showed that genes involved in hormone activity, receptor ligand activity, receptor regulator activity, signal receptor activator activity and signal receptor binding, which were upregulated in the L group compared to the C group, were significantly downregulated in the T group compared to the L group. These results suggest that taurine may act as an osmoregulator and antioxidant [49], participating in ion balance and ROS scavenging, thus reducing reliance on hormone and receptor signaling [50]. Moreover, genes involved in the responses to light and abiotic stimuli were also downregulated in the T group compared to the L group, suggesting that taurine can reduce the sensitivity of shrimp postlarvae to external stimuli [51], which could be responsible for the survival-promoting role of taurine seen in our experiment. On the other hand, taurine supplementation was correlated with the upregulation of bidirectional epithelial cell proliferation control, along with bidirectional changes in non-canonical Wnt signaling pathway components, suggesting a potential link between taurine and Wnt signaling pathway modulation, which might contribute to the reparative proliferation of the gill or intestinal epithelium [52] while preventing over-proliferation, leading to structural abnormalities [53]. Meanwhile, peptidyl-glutamine methylation and rRNA base methylation were also upregulated in the T group compared to the L group, which suggested that taurine may activate methyl metabolism to enhance the activity of osmoregulatory proteins [54]. Besides the GO enrichment, the KEGG enrichment analysis of the DEGs in the L and T groups suggested that taurine may activate processes involving carbohydrates (starch and sucrose metabolism), amino acids (beta-alanine metabolism, histidine metabolism, lysine degradation, arginine and proline metabolism), and vitamins (vitamin digestion and absorption) to adapt to low-salinity stress [55], which may be responsible for the growth-promoting roles of taurine in our study. Moreover, steroid hormone biosynthesis, which was upregulated in the L group compared to the C group, was downregulated in the T group, which may reflect taurine’s capacity to alleviate low-salinity stress in shrimp [56].

Finally, compared to the 737 differentially expressed genes between the C and L groups, only 529 differentially expressed genes (396 upregulated and 133 downregulated) were detected between the shrimp postlarvae in the low-salinity + taurine group and those in the control group. In *Channa argus*, the number of DEGs between starved and satiated specimens in the cultured group was much higher than that of DEGs between starved and satiated specimens in the wild group, indicating that cultured *C. argus* was more sensitive to feeding conditions [57]. Recent research has found that adding taurine to feed can improve the antioxidant capacity of fish [58], promote osmoregulation in marine bivalves [59] and reduce cortisol levels to alleviate stress in zebrafish [60]. Thus, the decreased DEGs between the C and T groups compared to those between the C and L groups suggested that taurine supplementation may help mitigate low-salinity-induced stress. However, the GO enrichment analysis of the DEGs between the C and T groups suggested that taurine upregulated genes involved in the regulation of developmental growth, cell development and cell projection organization, as well as neuron differentiation, nervous system development and neurogenesis, which is in accordance with previous studies reporting that taurine supplementation upregulated various developmental growth pathways [61]. Moreover, the downregulated cysteine-type endopeptidase activity found with GO enrichment and downregulated autophagy in the T group found with KEGG enrichment may suggest that taurine can suppress low-salinity-induced cell death, thereby maintaining cellular integrity [56].

## 5. Conclusions

In this study, low-salinity stress significantly restricted *L. vannamei* postlarva survival and caused histological changes, induced NKA overactivation, and differentially regulated gene expression. Taurine can serve as an osmolyte to effectively mitigate low-salinity stress-induced damage while simultaneously promoting epithelial cell proliferation in shrimp postlarvae. However, the present study was still limited by its short duration and lack of mechanistic validation. In future research, dose–response studies, longitudinal trials and proteomic validation of key targets are suggested to fully confirm the relevant regulatory mechanism.

## Data Availability

The RNA-seq data were deposited in the NCBI Sequence Read Archive (SRA) under the permanent accession number PRJNA1295440.

## Supplementary Materials

The following supporting information can be downloaded at: https://www.mdpi.com/article/10.3390/biology14081082/s1.

## References

[1] Mathan Muthu C.M., Vickram A.S., Bhavani Sowndharya B., Saravanan A., Kamalesh R., Dinakarkumar Y. (2024). A comprehensive review on the utilization of probiotics in aquaculture towards sustainable shrimp farming. Fish Shellfish Immunol..

[2] Islam M.M., Sarker A., Choudhury A., Ahmed N., Shafi A.A., Niloy N.T., Hossain M.S., Ali M.S., Chowdhury A., Ferdaus M.H. (2025). ShrimpDiseaseBD: An image dataset for detecting shrimp diseases in the aquaculture sector of Bangladesh. Data Brief..

[3] Hassan M., Elias N., Hassan M., Mocktar N., Harun N. (2025). Integrated overview on status, diagnosis and disease management of Acute Hepatopancreatic Necrosis Disease (AHPND) in shrimp aquaculture through metallic nanoparticles (MNPs) application—A review. Aquaculture.

[4] Pimentel O., Schwarz M., Senten J., Wasielesky W., Urick S., Carvalho A., McAlhaney E., Clarington J., Krummenauer D. (2025). The super-intensive culture of *Penaeus vannamei* in low salinity water: A comparative study among recirculating aquaculture system, biofloc, and synbiotic systems. Aquaculture.

[5] Havird J.C., Santos S.R., Henry R.P. (2014). Osmoregulation in the Hawaiian anchialine shrimp *Halocaridina rubra* (Crustacea: Atyidae): Expression of ion transporters, mitochondria-rich cell proliferation and hemolymph osmolality during salinity transfers. J. Exp. Biol..

[6] Henry R.P., Lucu C., Onken H., Weihrauch D. (2012). Multiple functions of the crustacean gill: Osmotic/ionic regulation, acid-base balance, ammonia excretion, and bioaccumulation of toxic metals. Front. Physiol..

[7] Giffard-Mena I., Ponce-Rivas E., Sigala-Andrade H.M., Uranga-Solís C., Re A.D., Díaz F., Camacho-Jiménez L. (2024). Evaluation of the osmoregulatory capacity and three stress biomarkers in white shrimp *Penaeus vannamei* exposed to different temperature and salinity conditions: Na+/K+ ATPase, Heat Shock Proteins (HSP), and Crustacean Hyperglycemic Hormones (CHHs). Comp. Biochem. Physiol. B Biochem. Mol. Biol..

[8] Cao L., Xiong S., Wu Z., Ding L., Zhou Y., Sun H., Zhu M., Lee W.T., Nie X., Bian J.S. (2021). Anti-Na+/K+-ATPase immunotherapy ameliorates α-synuclein pathology through activation of Na+/K+-ATPase α1-dependent autophagy. Sci Adv..

[9] Lucu C., Towle D.W. (2003). Na(+)+K(+)-ATPase in gills of aquatic crustacea. Comp. Biochem. Physiol. A Mol. Integr. Physiol..

[10] Diwan A.D., Harke S.N., Panche A. (2022). Biological mechanism of osmoregulatory stress in penaeid shrimp, *Penaeus indicus*. J. Proteom. Bioinform.

[11] Venkitaraman P.R., Jayalakshmy K.V., Abhilash K.R. (2013). Effect of eyestalk extirpation on haemolymph ionic concentration of *Metapenaeus monoceros*. J. Exp. Biol. Agric. Sci..

[12] Diwan A.D., Laxminarayana A. (1989). Osmoregulatory ability of Penaeus indicus H Milne Edwards in relation to varying salinities. J. Anim. Sci..

[13] van der Bosch de Aguilar P. (1976). Neurosecretion and hydroelectrolytic regulation in *Artemia salina* (author’s transl). Experientia.

[14] Abe H., Yoshikawa N., Sarower M.G., Okada S. (2005). Physiological function and metabolism of free D-alanine in aquatic animals. Biol. Pharm. Bull..

[15] de Faria S.C., Augusto A.S., McNamara J.C. (2011). Intra- and extracellular osmotic regulation in the hololimnetic Caridea and Anomura: A phylogenetic perspective on the conquest of fresh water by the decapod Crustacea. J. Comp. Physiol. B.

[16] Yang Y., Ni J., Niu D., Zheng G., Li Y. (2024). Physiological response of the razor clam Sinonovacula constricta exposed to hyposalinity stress. Aquac. Fish..

[17] Salze G.P., Davis D.A. (2015). Taurine: A critical nutrient for future fish feeds. Aquaculture.

[18] Han J., Kim E., Ho W.K., Earm Y.E. (1996). Blockade of the ATP-sensitive potassium channel by taurine in rabbit ventricular myocytes. J. Mol. Cell Cardiol..

[19] Laplante M., Sabatini D.M. (2012). mTOR signaling in growth control and disease. Cell.

[20] Yue Y.R., Liu Y.J., Tian L.X., Gan L., Yang H., Liang G., He J. (2013). The effect of dietary taurine supplementation on growth performance, feed utilization and taurine contents in tissues of juvenile white shrimp (*Litopenaeus vannamei*, Boone, 1931) fed with low-fishmeal diets. Aquac. Res..

[21] Li H., Huang X., Wang X., Yan M., Zheng X. (2017). Effect of dietary taurine supplementation on the growth, body composition, digestive enzyme activity and anti-stress ability of *Litopenaeus vannamei* in freshwater culture. J. Shanghai Ocean. Univ..

[22] Mai H., Li Y., Song Z., Zeng Y., Lin P., Sun Z., Mai K., Tan B., Ye C. (2025). Effect of taurine on growth and immune response of Pacific white shrimp (*Litopenaeus vannamei*) cultured at different temperatures. Aquaculture.

[23] Zhang D., Wang F., Dong S., Lu Y. (2016). De novo assembly and transcriptome analysis of osmoregulation in *Litopenaeus vannamei* under three cultivated conditions with different salinities. Gene.

[24] Rahi M., Moshtaghi A., Mather P.B., Hurwood D.A. (2018). Osmoregulation in decapod crustaceans: Physiological and genomic perspectives. Hydrobiologia.

[25] Re A.D., Díaz F., Ponce-Rivas E., Giffard I., Munoz-Marquez M.E., Sigala-Andrade H.M. (2012). Combined effect of temperature and salinity on the thermotolerance and osmotic pressure of juvenile white shrimp *Litopenaeus vannamei* (Boone). J. Therm. Biol..

[26] Pan L.Q., Luan Z.H., Jin C.X. (2006). Effects of Na+/K+ and Mg2+/Ca2+ ratios in saline groundwaters on Na^+^–K^+^-ATPase activity, survival and growth of *Marsupenaeus japonicus* postlarvae. Aquaculture.

[27] Walker S.J., Neill W.H., Lawrence A.L., Gatlin D.M. (2009). Effect of salinity and body weight on ecophysiological performance of the Pacific white shrimp (*Litopenaeus vannamei*). J. Exp. Mar. Biol. Ecol..

[28] Chong-Robles J., Charmantier G., Boulo V., Lizárraga-Valdéz J., Enríquez-Paredes L.M., Giffard-Mena I. (2014). Osmoregulation pattern and salinity tolerance of the white shrimp *Litopenaeus vannamei* (Boone, 1931) during post-embryonic development. Aquaculture.

[29] Hu D., Pan L., Zhao Q., Ren Q. (2015). Transcriptomic response to low salinity stress in gills of the Pacific white shrimp, *Litopenaeus vannamei*. Mar. Genomics.

[30] Lu H., Chen W., Peng K., Huang M., Zhao J., Chen X., Sun Y., Ruan Z., Li C., Liu D. (2023). Rapid adaptive and acute stimulatory responses to low salinity stress in Pacific white shrimp (*Litopenaeus vannamei*): Insights from integrative transcriptomic and proteomic analysis. Comp. Biochem. Physiol. Part D Genom. Proteom..

[31] Yancey P.H. (2005). Organic osmolytes as compatible, metabolic and counteracting cytoprotectants in high osmolarity and other stresses. J. Exp. Biol..

[32] Takeuchi K., Toyohara H., Kinoshita M., Sakaguchi M. (2000). Ubiquitous increase in taurine transporter mRNA in tissues of tilapia, *Oreochromis mossambicus* during high-salinity adaptation. Fish. Physiol. Biochem..

[33] Xu H., Liu T., Feng W., He J., Han T., Wang J., Wu Q., Wang C. (2024). Evaluation of the dietary taurine requirement for early juvenile mud crab *Scylla paramamosain*. Aquaculture.

[34] Velselvi R., Dasgupta S., Varghese T., Sahu N.P., Tripathi G., Panmei H., Singha K.P., Krishna G. (2021). Taurine and/or inorganic potassium as dietary osmolyte counter the stress and enhance the growth of GIFT reared in ion imbalanced low saline water. Food Chem.

[35] Thiruvasagam T., Felix N., Nazir M.I., Ranjan A., Prabu E. (2024). Enhanced cholesterol utilization and impact on growth through taurine supplementation in high plant-based diets of Pacific white shrimp, *Penaeus vannamei* in biofloc system. Anim. Feed. Sci. Tech..

[36] Schuller-Levis G.B., Park E. (2004). Taurine and its chloramine: Modulators of immunity. Neurochem. Res..

[37] Zheng Z., Chen Y., Huang K., Li D., Gao Z., Yan X., Huo Z., Qin Y. (2025). Taurine promotes the rapid recovery of clams (*Ruditapes philippinarum*) after aerial exposure through the glutathione pathway and by inhibiting apoptosis. Aquac. Rep..

[38] Apell H.J., Hitzler T., Schreiber G. (2017). Modulation of the Na,K-ATPase by Magnesium Ions. Biochemistry.

[39] Lovett D.L., Verzi M.P., Burgents J.E., Tanner C.A., Glomski K., Lee J.J., Towle D.W. (2006). Expression profiles of Na^+^,K^+^-ATPase during acute and chronic hypo-osmotic stress in the blue crab *Callinectes sapidus*. Biol. Bull..

[40] Sun H., Zhang L., Ren C., Chen C., Fan S., Xia J.J., Lin H., Hu C. (2011). The expression of Na, K-ATPase in *Litopenaeus vannamei* under salinity stress. Mar. Biol. Res..

[41] Sun H., Dong J., Ren C., Zhang L., Dan C., Chen C., Zhang Y., Hu C. (2015). Cloning and differential expression of Na, K-ATPase in *Penaeus vannamei*. Mar. Biol. Res..

[42] Hurtado M.A., Racotta I.S., Civera R., Ibarra L., Hernández-Rodríguez M., Palacios E. (2007). Effect of hypo—and hypersaline conditions on osmolality and Na+/K+-ATPase activity in juvenile shrimp (*Litopenaeus vannamei*) fed low—and high-HUFA diets. Comp. Biochem. Physiol. A Mol. Integr. Physiol..

[43] Leil T.A., Chen Z.W., Chang C.S., Olsen R.W. (2004). GABAA receptor-associated protein traffics GABAA receptors to the plasma membrane in neurons. J. Neurosci..

[44] Geahchan S., Baharlouei P., Rahman A. (2022). Marine Collagen: A Promising Biomaterial for Wound Healing, Skin Anti-Aging, and Bone Regeneration. Mar. Drugs.

[45] Le G., Yang L., Du H., Hou L., Ge L., Sylia A., Muhmood A., Chen X., Han B., Huang K. (2022). Combination of zinc and selenium alleviates ochratoxin A-induced fibrosis via blocking ROS-dependent autophagy in HK-2 cells. J. Trace Elem. Med. Biol..

[46] Ahearn G.A., Duerr J.M., Zhuang Z., Brown R.J., Aslamkhan A., Killebrew D.A. (1999). Ion transport processes of crustacean epithelial cells. Physiol. Biochem. Zool..

[47] Zhu G., Lu K., Lai Y., Wang L., Wang F., Li N., Peng Y., Gong H. (2024). Effects of dietary 25-hydroxyvitamin D3 on growth, calcium-phosphorus metabolism, lipid metabolism and immunity of *Litopenaeus vannamei* at low salinity. Aquac. Rep..

[48] Guldenpfennig C., Teixeiro E., Daniels M. (2023). NF-kB’s contribution to B cell fate decisions. Front. Immunol..

[49] Chiang J.Y. (2013). Bile acid metabolism and signaling. Compr. Physiol..

[50] Jong C.J., Azuma J., Schaffer S. (2012). Mechanism underlying the antioxidant activity of taurine: Prevention of mitochondrial oxidant production. Amino Acids.

[51] Ripps H., Shen W. (2012). Review: Taurine: A “very essential” amino acid. Mol. Vis..

[52] Lee S., Shin J.Y., Kwon O.S., Jun S.H., Kang N.G. (2024). Taurine and Polyphenol Complex Repaired Epidermal Keratinocyte Wounds by Regulating IL8 and TIMP2 Expression. Curr. Issues Mol. Biol..

[53] Du G., Liu Z., Yu Z., Zhuo Z., Zhu Y., Zhou J., Li Y., Chen H. (2021). Taurine represses age-associated gut hyperplasia in Drosophila via counteracting endoplasmic reticulum stress. Aging Cell.

[54] Redmond H.P., Stapleton P.P., Neary P., Bouchier-Hayes D. (1998). Immunonutrition: The role of taurine. Nutrition.

[55] Ribeiro R.A., Bonfleur M.L., Batista T.M., Borck P.C., Carneiro E.M. (2018). Regulation of glucose and lipid metabolism by the pancreatic and extra-pancreatic actions of taurine. Amino Acids.

[56] Li S., Wang D., Zhang M., Zhang C., Piao F. (2022). Taurine Ameliorates Apoptosis via AKT Pathway in the Kidney of Diabetic Rats. Adv. Exp. Med. Biol..

[57] Jin X., Chen X., Guo G., Sun L., Wu X., Lin Y., Niu X., Kong Y., Li M., Wang G. (2025). Brain transcriptome analysis of snakehead (*Channa argus*) under starvation and satiation conditions and identification of differentially expressed gene response to feeding regulation. Aquac. Rep..

[58] Suehs B., Gatlin III D.M. (2021). Evaluating the dietary taurine requirement of hybrid striped bass (*Morone Chrysops* × *M. Saxatilis*). Aquaculture.

[59] Chen Y., Dong M., Dong Z., Li Y., Niu D. (2024). Function of taurine and its synthesis-related genes in hypertonic regulation of *Sinonovacula constricta*. Comp. Biochem. Physiol. A Mol. Integr. Physiol..

[60] Mezzomo N.J., Fontana B.D., Müller T.E., Duarte T., Quadros V.A., Canzian J., Pompermaier A., Soares S.M., Koakoski G., Loro V.L. (2019). Taurine modulates the stress response in zebrafish. Horm. Behav..

[61] Shao X., Hu Z., Hu C., Bu Q., Yan G., Deng P., Lv L., Wu D., Deng Y., Zhao J. (2012). Taurine protects methamphetamine-induced developmental angiogenesis defect through antioxidant mechanism. Toxicol. Appl. Pharmacol..

