# Supplementary material for: Taurine Supplementation Enhances the Resistance of Litopenaeus vannamei Postlarvae to Low-Salinity Stress
Source: Biology (Basel). 2025 Aug 19;14(8):1082. doi: 10.3390/biology14081082 (PMC12383651; doi:10.3390/biology14081082)
Supplement: Supplementary file 1 [file biology-14-01082-s001.zip › biology-3751067 Supplementary Table S1&S2.pdf]

Supplementary Materials

Supplementary Table S1. Primers used in the present study.

Gene	Forward Sequence	Reverse Sequence
NSA2	GCCGCAGAACGAGCATATTG	CTCCTCATGGGCTTTCAGGG
yki	CTGCAGACTCCAGCTTCGTT	CTCAACCTGAAGGCGCTGTA
DAO5	TCGACCAAATGGACAGCGAA	CGCGTTCAGCTACACTCAGA
CryA2	AAAGTGGCCAACCAGGACAA	TCTTGCCCTGCATTCTGAGG
ChB2	ACATCCAGTTCACGTCCAGC	GCTGGGTACTCGCTAAGGAC
Cte3	ATCAACCAGCCACAGAGTGC	ATCGTCCGATTCCCAGAGGA
CruC1	AGCCTTTCGCTTGACGGC	TTGTAGCAGTCGTCGCAGAG
I-Cy9	GGTGGCCACATGAGAGGAAA	GCTCGGACAATACTGGGTCC
PIG-M	CTGGCATGGATGCTTTTGCC	AGCACTGCCACGTGTTGATA
AMP1A	ACGGCATTGTCCTGTCTCAG	TCGAGGACGAACTGAGGGAT
Thh	AACTCCGACACGGACGTAAC	CTCCGGCGTTTGGTTCTTTG
POD2	GAGCTGATTTGGGTAGCGGT	CTCTGGTAGGCGCACGAAAA

Supplementary Table S2. Summary of the transcriptomic sequencing data collected from *L. vannamei* postlarvae in response to low salinity and taurine supplementation

Sample	Raw reads	Clean reads	Q30(%)	GC content(%)
Control(C)	87316458	87004990	97.08	44.82
Low-salinity(L)	87925440	87633350	97.16	46.11
Low-salinity + Taurine(T)	87519644	87189266	97.02	45.03
